# Domestic

**Published:** 1841-12-11

**Authors:** 


### DOMESTIC.

The following remarks are found under the editorial head in the New York Medical Gazette for Dec. 1st:

"We commence to-day the publication of Dr. John Augustine Smith's address at the opening of the present session of the College of Physicians and Surgeons. The friends of the Institution will be glad to hear that it is prospering; the number of students is greater than it has been for many years, and is still increasing. In another important respect, the Institution is more highly favoured than it has been, in the possession of the confidence and good will of the profession in New York. We may say of the whole profession, for we confidently believe that the College stands higher in the good opinion, has a larger measure of the respect, and a stronger hold on the good will of the profession, than it has had for years. In this the Institution, and those who control its destinies, reap the just reward of the high and honourable course which they have pursued under the peculiar circumstances in which they have been placed. Under many temptations, and with many excuses for doing otherwise, the College has never deviated from its course of quiet, unobtrusive usefulness; in such a course, the profession—the whole profession among whom it is situated, who, by their position, have the best opportunities, and by their characters are best qualified to judge of its merits—will sustain it. An Institution which has such support, may well dispense with all sinister arts; it needs no such aid, for when its friends are inquired of as to its character and pretensions, they may fearlessly reply, '*Ask the Profession in New York.*' "

The great city of New York is unquestionably rich in resources for medical teaching, and many districts of the state of which she is the capital, stand high in practical intelligence, perhaps *beyond any other section of the Union*; yet she has always been, *and still is*, peculiarly unfortunate in calling out those resources in such a manner as to render them available. We have watched her struggles, with regret at their failure, in years long gone by, and have been led to doubt whether there was not something in the very atmosphere of the great Commercial Emporium which unfitted it for the seat of medical instruction on an extended scale.

All cosmopolitan as our principles are, nothing would give us more pleasure than to have this doubt effectually dissipated. The first object in an attempted medical reform should be the establishment of unanimity and close bonds of brotherhood in the profession, giving the medical character that weight in the community to which it is justly entitled. The second, should be the direction of the whole energy of the profession, moving in solid phalanx, to the defence of the principle of *well regulated medical representation* in the immediate government of medical institutions—a principle neglected with us, as in our sister city, in consequence of a fundamental error in our forefathers, who established our principal colleges upon a foreign model, ill adapted to the genius of our political institutions. Next follows—"though last, not least"—the necessity of taking ample time to accomplish great results. There has been generally too much parade, or too much vastness of scheme, in the medical movements of New York, to place her where she ought to be—*among* the principal centres of medical instruction. Had half the real wisdom been displayed in her professional legislation, that she has shown in the foundation of her noble primary schools and academies, *some years of well-regulated and staid exertion* might supply her with the material for a medical college, or colleges, of the highest rank in point of numbers. The "course of quiet, unobtrusive use fulness," pursued by the College of Physicians and Surgeons, is calculated to bring about such a result in time, as it would with any similar institution in the same happily located city, provided the profession and the legislature could be brought to act in concert, and the college itself should be disposed to advance and expand with the age, adopting the motto "Festina lente!" To hear that it is disposed to do so, and that it already begins to meet a "just reward," will give pleasure to every true friend of medicine throughout the Union.

